# Lipopolysaccharide significantly influences the hepatic triglyceride metabolism in growing pigs

**DOI:** 10.1186/s12944-015-0064-8

**Published:** 2015-06-30

**Authors:** Zhiqing Liu, Weifeng Liu, Yanping Huang, Jun Guo, Ruqian Zhao, Xiaojing Yang

**Affiliations:** Key Laboratory of Animal Physiology & Biochemistry, Nanjing Agricultural University, Nanjing, 210095 People’s Republic of China

**Keywords:** LPS, Growing pigs, Triglyceride metabolism, Glucocorticoid receptor

## Abstract

**Background:**

In the practical commercial pig farms, inflammation is a perennial problem, yet most of studies on inflammation are focused on immune response. Actually, inflammation can induce body metabolism disorder which will finally influence animals’ growth. In this study, we investigated the effect of acute inflammation on the triglyceride (TG) metabolism in the liver of growing pigs and the possible underlying mechanisms.

**Methods:**

Twelve male growing pigs were randomly divided into two groups, a control group (received saline) and a LPS group (intramuscular injected with 15 μg/kg LPS). Six hours after LPS injection, the pigs were euthanized and sampled. Biochemical indexes, inflammation factors, lipid metabolism related parameters and mitochondrial function were evaluated. The relationship between glucocorticoid receptor (GR) and the key enzymes of *de novo* lipogenesis were also investigated by chromatin immunoprecipitation assay (ChIP).

**Results:**

LPS induced a serious inflammation in the liver of growing pigs proved by liver morphologic changes, the up-regulated plasma cortisol, tumor necrosis factor-α (TNF-α) content and gene expression of inflammation related genes in liver. For *de novo* lipogenesis, LPS significantly decreased the gene expression of fatty acid synthase (FAS), Acetyl-CoA carboxylase-1 (ACC-1) and Stearoyl-CoA desaturase-1 (SCD-1), and the protein expression of ACC-1 and SCD-1. For lipolysis, only the gene expression of adipose triglyceride lipase (ATGL) was decreased. LPS did nothing to the gene expression of hormone-sensitive lipase (HSL) and the lipolytic enzymes activities. For β-oxidation, LPS significantly increased the protein expression of CPT-1α, but the gene expression of mitochondrial DNA-encoded genes and the activities of mitochondrial complex IV and V demonstrated no obviously changes. Furthermore, ChIP results showed that LPS significantly decreased the level of GR binding to ACC-1 promoter.

**Conclusion:**

LPS infection has a profound impact on hepatic TG metabolism. This impact is mainly demonstrated by the significantly deceased *de novo* lipogenesis, and GR may involve in its regulation.

## Background

In the clinical practice of modern intensive farms, pigs are often exposure to various harmful microbes and the inflammation easily exists. However, most of investigations concerning infection in pigs were focused on the changes of immune response and organ pathology [1, 2]. Actually, the host response to infection is usually associated with multiple disturbances in intermediary metabolism which will finally result in animal growth retard or decreased products quality. Therefore, understanding the metabolism changes induced by inflammation is vital to livestock husbandry.

Triglyceride (TG) is extremely essential for growing pigs except as energy storage [3]. It participates in various functions, including structure, signaling and, thermal insulation [4], and functions as a deposit for essential and non-essential fatty acids [5]. The liver is the main site of TG metabolism [6]. It has been reported that the impaired TG metabolism is association with many hepatic diseases, including non-alcoholic steatosis, steatohepatitis, fibrosis, cirrhosis and cancer in human and rodent models [7, 8]. However, how the TG metabolism changes in growing pigs after infection are still largely unclear.

TG metabolism composes by synthesis (*de novo* lipogenesis) and catabolism (lipolysis and ß-oxidation). Acetyl-CoA carboxylase-1 (ACC-1), Fatty acid synthase (FAS) and stearoyl-CoA desaturase-1 (SCD-1) are key enzymes of *de novo* lipogenesis. Hormone-sensitive lipase (HSL) and adipose TG lipase (ATGL) are the enzymes that catalyze the rate limiting hydrolysis step [9]. For ß-oxidation, carnitine palmitoyltransferase-1α (CPT-1α) serves as a key regulator and transports the fatty acid into mitochondria [10]. Mitochondria function will finally influence the fatty acid oxidation. If inflammation influences the porcine hepatic lipid metabolism, which step of lipid metabolism can be affected is unknown.

Meanwhile, inflammation can induce a significant increase of endogenous glucocorticoid level, and the previous studies confirm a link between the increased glucocorticoid and TG metabolism in mice and chicken [11, 12]. At the same time, glucocorticoid receptor (GR) plays a significant role in the anti-inflammatory effect of glucocorticoid on target tissues [13]. Whether the alteration of TG metabolism, if changed, is correlated with the GR in the liver of growing pigs is also unknown. Therefore, in the present study, we investigated the effect of acute inflammation on the TG metabolism in the liver of growing pigs after the injection with lipopolysaccharide (LPS), and unravel the possible underlying mechanisms. The results offer a clue to understand the hepatic metabolism after inflammation in pigs.

## Results

### The effect of LPS on plasma parameters

LPS significantly increased plasma aspartate aminotransferase (AST), cortisol and tumor necrosis factor-α (TNF-α) content but not alanine aminotransferase (ALT) content compared to Control (Con) group. LPS didn’t influence TG content in plasma compared to Con group (Table 1).

**Table 1:** Concentrations of metabolites in plasma

Parameter	Con	LPS
ALT in plasma(U/L)	46.78 ± 1.86	37.22 ± 9.9
AST in plasma(U/L)	99.89 ± 16.9	172.89 ± 22.22^*^
Cortisol in plasma (μg/L)	35.85 ± 2	108.25 ± 24.27^*^
TNF-α in plasma (fmol/L)	4.03 ± 0.24	7.48 ± 0.46^*^
TG in plasma (μmol/L)	855 ± 68.06	731.67 ± 156.38

Results are presented as mean ± SEM

^*^ *P* < 0.05 versus Con

### The effect of LPS on liver morphologic changes and inflammation pathways

**Fig. 1:**
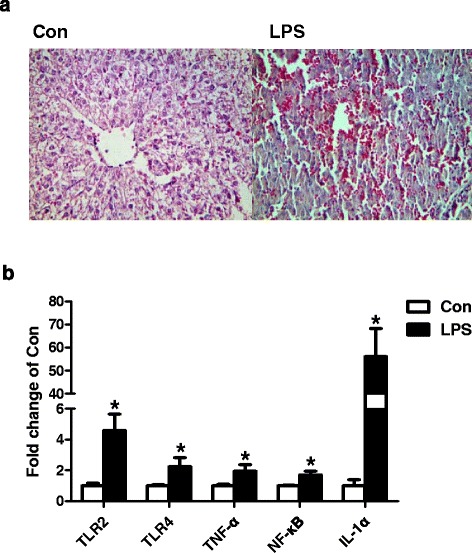
Liver morphologic changes (**a**) and the gene expression of TLR2, TLR4, TNF-α, NF-κB, IL-1α (**b**) in Con and LPS groups. ^*^P < 0.05 versus Con

The hematoxylin and eosin staining revealed that the morphology changes of liver tissues after LPS injection compared to the control pigs. After LPS treatment, the heptocytes arranged extremely disordered with unclear nucleus, while the cytoplasmic staining was uneven, and a large number of red blood cells overflowed (Fig. 1a). For inflammation related genes expression, LPS significantly increased the expression of toll-like receptor 2 (TLR2), toll-like receptor 4 (TLR4), TNF-α, nuclear factor kappa B (NF-κB) and interleukin-1α (IL-1α) in liver (Fig. 1b).

### The effect of LPS on de novo lipogenesis in liver

**Fig. 2:**
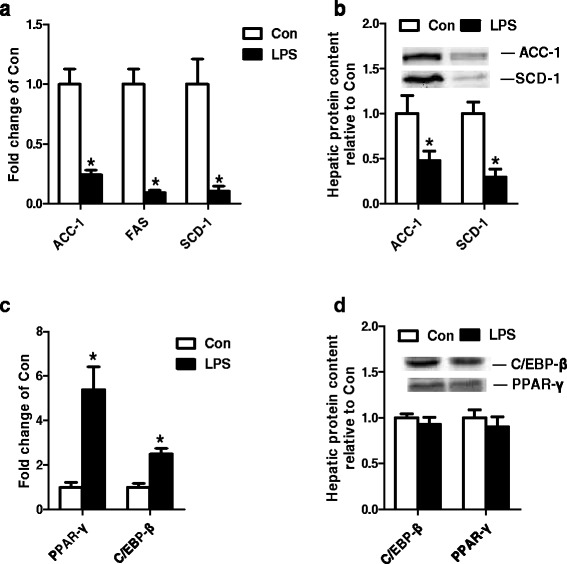
The gene expression of ACC-1, FAS, SCD-1 (**a**), the protein expression of ACC-1 and SCD-1 (**b**), the gene expression of PPAR-γ and C/EBP-β (**c**), the protein expression of PPAR-γ and C/EBP-β (**d**) in Con and LPS groups. ^*^ *P* < 0.05 versus Con

LPS dramatically decreased the gene expression of ACC-1, FAS and SCD-1 compared with Con group (Fig. 2a). Consistent with gene expression, LPS also significantly decreased the protein expression of ACC-1 and SCD-1 (Fig. 2b). For transcription factors of lipid synthesis, LPS significantly increased the gene expression of CEBP-β and PPAR-γ (Fig. 2c). But there is no difference in protein expression of CEBP-β and PPAR-γ between Con and LPS group (Fig. 2d).

### The effect of LPS on lipolysis and β-oxidation in liver

**Fig. 3:**
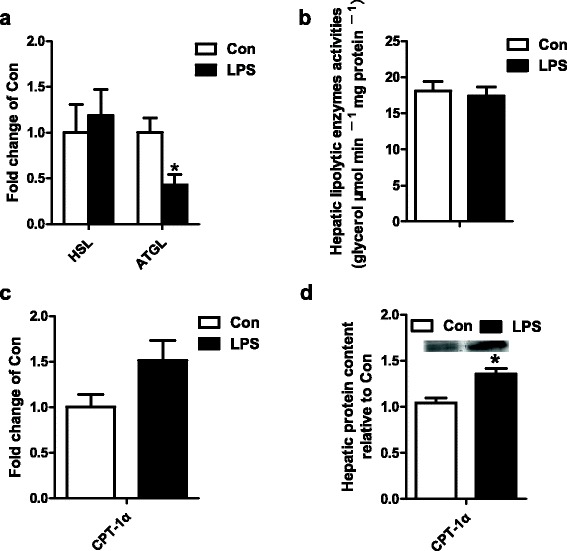
The gene expression of HSL and ATGL (**a**), the hepatic lipolytic enzymes activities (**b**), the gene expression of CPT-1α (**c**), the protein expression of CPT-1α (**d**) in Con and LPS groups. ^*^ *P* < 0.05 versus Con

For lipolysis, the gene expression of adipose TG lipase (ATGL) was significantly decreased by LPS compared to Con group (Fig. 3a). But LPS did nothing to the gene expression of hormone-sensitive lipase (HSL) and the lipolytic enzymes activities (Fig. 3a and b). For β-oxidation, LPS tended to increase the gene expression of CPT-1α (*p* = 0.073) compared to Con. And the protein expression of CPT-1α was significantly increased by LPS compared to Con group (Fig. 3c and d).

### The effect of LPS on mitochondrial function

**Fig. 4:**
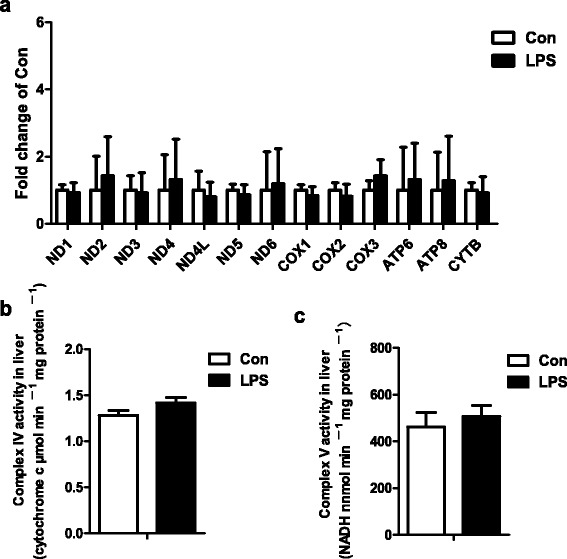
The gene expression of mitochondrial DNA-encoded genes (**a**) and the activities of mitochondrial complex IV (**b**) and V (**c**) in Con and LPS groups. ^*^ *P* < 0.05 versus Con

LPS did not change the gene expression of mitochondrial DNA-encoded genes. The gene expression of NADH dehydrogenase subunit 1 (ND1), NADH dehydrogenase subunit 2 (ND2), NADH dehydrogenase subunit 3 (ND3), NADH dehydrogenase subunit 4 (ND4), NADH dehydrogenase subunit 4 L (ND4L), NADH dehydrogenase subunit 5 (ND5), NADH dehydrogenase subunit 6 (ND6), cytochrome C oxidase subunit 1 (COX1), cytochrome C oxidase subunit 2 (COX2), ATP synthase F0 subunit 6 (ATP6), ATP synthase F0 subunit 8 (ATP8) and cytochrome b (CYTB) all showed no difference between Con group and LPS group. The gene expression of cytochrome oxidase subunit 3 (COX3) was tend to be up-regulated (*p* = 0.09) by LPS compared to Con group (Fig. 4a). Consistent with gene expression, LPS had no effect on mitochondrial complex IV and V activities (Fig. 4b and c).

### The effect of LPS on the level of GR binding to ACC-1 promoter in liver

**Fig. 5:**
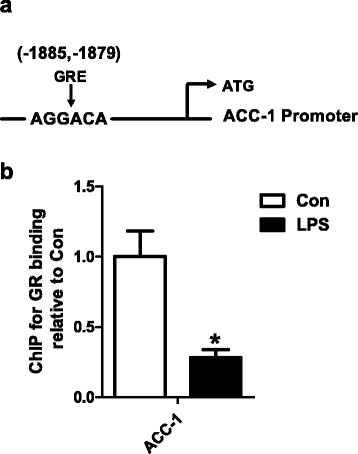
The location of glucocorticoid receptor element (GRE) on the ACC-1 promoter (**a**), the level of glucocorticoid receptor (GR) bingding to ACC-1 promoter (**b**) in Con and LPS groups. ^*^ *P* < 0.05 versus Con

LPS significantly decreased the level of GR binding to ACC-1 promoter in liver compared to Con group (Fig. 5b).

## Discussion

The hepatic TG disorder is a major health problem, and much attention has been paid on its pathogenesis and etiology [14, 15]. The disorder can influence the liver function and animals’ growth. However, for growing pigs under inflammation, most of previous studies focus on the immune response [1, 2]. There is no study until now focused on the hepatic TG changes in growing pigs after infection. Results form the present study showed that LPS induced a dramatic effect on hepatic TG metabolism of growing pigs, and the effect mainly on *de novo* lipogenesis. Moreover, GR is partly involved in this process.

Consistent with the results of previous researches on liver injuries induced by LPS [16, 17], in this experiment, LPS also induced serious liver injury demonstrated by morphology changes and up-regulated AST level. It has been shown that endotoxin can active macrophages and induce the expression of inflammation related genes in the liver of numerous models [18–20]. In the present study, LPS significantly increased the plasma cortisol and TNF content, and the gene expression of TLR2, TLR4, TNF-α, NF-κB and IL-1α in liver also showed significantly increase. The results indicated LPS treatment led to the hepatic local inflammation and acute injury.

Inflammation is usually associated with multiple disturbances in intermediary metabolism, including TG metabolism. In mice model, it has been demonstrated that the content of serum TG in endotoxin-poisoned mice is decreased slightly 2 h postintoxication compared with Con group. Thereafter, the level of serum TG in poisoned mice changed to elevation, and markedly increased after 12–24 h [21]. In the present study, TG content in plasma had no change 6 h after LPS treatment in growing pigs. In subsequently time, the plasma TG content may increase in LPS group compared to Con group.

The liver plays a key role in TG metabolism. Hepatic TG metabolism disorder is now understood to have an important action in the development of advanced liver disease [22, 23]. So we investigated the effect of acute inflammation on the TG metabolism in the liver of growing pigs. For hepatic *de novo* lipogenesis, the gene expression of ACC-1, FAS SCD-1 and protein content of ACC-1 and SCD-1 were significantly decreased by LPS in our study. It was consistent with previous studies in other tissues [24–26]. C/EBP-β and PPAR-γ are well-established transcription factors involved in lipid metabolism during adipogenesis [27]. Excepted as lipid transcription factor, it has been shown that these two genes are involved in the anti-inflammatory regulation. Previous studies demonstrated that C/EBP-β could be activated by LPS in osteoblasts [28] and the live of rat [29]. Meanwhile, LPS also increased the expression of PPAR-γ in rat brain [30]. Consistent with the previously reports, in the present study, the gene expression of C/EBP-β and PPAR-γ in porcine liver increased significantly, while the protein expression of both C/EBP-β and PPAR-γ demonstrated no difference. Whatever, given the important role of C/EBP-β and PPAR-γ in adipogenesis, it is possible that the aberrant lipid accumulation will come out in subsequently time.

Lipolysis is the breakdown of lipids and involves hydrolysis of TG into glycerol and free fatty acids. In adipose tissue, previous study showed that HSL activity in poisoned mice increased appreciably 2 h after injection, but after 18 h, it was less than control mice [21]. In our study, it seems that LPS had no obviously effect on lipolysis which evidenced by the unaffected gene expression of HSL and the activities of the lipolytic enzymes. It is well-known that fatty acid degradation includes three major steps: activation and transport into the mitochondria, β-oxidation and electron transport chain. Fatty acids are transported across the outer mitochondrial membrane by CPT-1α, which is believed to be the rate-limiting step in fatty acid oxidation [10]. Previous study showed that the activity and protein expression of CPT-1α was significantly increased in LPS induced acute liver failure in rats [31]. Also, in our study, CPT-1α protein expression was increased in growing pigs. Beta-oxidation is to generate acetyl-coA, which enters the citric acid cycle, and NADH and FADH2, which are used by the electron transport chain. So, we investigated the mitochondrial function. Previous study showed that mitochondrial complex IV were not altered in rat brain and liver after sepsis [32, 33]. Our results showed that, in growing pigs, LPS did not change the gene expression of mitochondrial DNA-encoded genes, and the mitochondrial complex IV and V activities didn’t demonstrate significantly difference. Anyway, the state of fatty acid catabolism in the mitochondria still need further investigate. All the results above indicated that the effect of LPS on the hepatic TG metabolism of growing pigs are mainly on *de novo* lipogenesis that significantly down-regulated.

Glucocorticoid receptor (GR) is the receptor for glucocorticoid and also acts as a transcription factor for distinct target genes through both direct DNA binding and protein–protein interactions with other transcription factors [34]. In the present study, the level of corticosteroid increased significantly after LPS infection, and the gene and protein level of GR were significantly decreased (date was shown in other unpublished paper). So we try to investigate the relationship between GR and the key enzymes of *de novo* lipogenesis. Previous study suggests that there is glucocorticoid receptor element (GRE) lies within the 2.1-kb region upstream from transcription start site in the rat FAS gene [35]. GRE in or nearby the mouse ACC-1 and SCD-1 genes in 3 T3-L1 mouse adipocytes were also identified by chromatin immunoprecipitation sequencing (ChIPseq) [36]. In our study, we predict the GREs in the promoters of them: ACC-1 is “AGGACA” (−1885, −1879), FAS is “AGAACA” (−1082, −1076), SCD-1 are “TGTTCT” (−1678, −1672) and “TGTACA” (−990, −984). The ChIP results showed that LPS significantly decreased the level of GR binding to ACC-1 promoter in the livers of growing pigs compared to Con group. But GR is not binding on the promoter of FAS and SCD-1. The results show that GR is partly involved in the effect of inflammation on *de novo* lipogenesis.

## Conclusions

In conclusion, LPS have a profound effect on hepatic TG metabolism. And the effect is mainly on *de novo* lipogenesis genes which are significantly down-regulated. GR may involve in the process.

## Methods

### Ethics statement

The experiment was conducted following the guidelines of Animal Ethics Committee at Nanjing Agricultural University, China. The euthanasia and sampling procedures complied with the “Guidelines on Ethical Treatment of Experimental Animals” (2006) No. 398 set by the Ministry of Science and Technology, China and “the Regulation regarding the Management and Treatment of Experimental Animals” (2008) No. 45 set by the Jiangsu Provincial People’s Government.

### Animals and experimental design

Twelve (Duroc × Landrace × Large White) male growing pigs with the average body weight of 12 ± 0.5 kg were used in this experiment, which were randomly divided into two groups, a control group and a LPS group. Pigs were fed 3 times a day with a commercial diet. Water was available ad libitum. After 1 week adaption, the LPS group pigs were intramuscular injected with 2 mL LPS (E.Coliserotype, Sigma Aldrich Ireland.Ltd, Dubin, Ireland ) at a dose of 15 μg/kg bodyweight, while the control received the same volume of saline. Six hours after LPS injection, the pigs were euthanized and sampled. 10 mL blood was collected into tubes containing EDTA-Na2, then centrifuged at 3500 rpm for 10 min for plasma collecting. Small portions of livers were taken within 20 min postmortem and immediately frozen in liquid nitrogen. The plasma and liver was stored at −20 °C and −80 °C respectively until further analysis. Liver samples for histochemical analysis were kept in 10 % formaldehyde.

### Hematoxylin and eosin staining

After being dehydrated through an ethanol-xylene series, the liver specimens were embedded in paraffin. Sections were cut (5 μm thick) and mounted on slides, each section was de-waxed in xylene and dehydrated with a graded alcohol series. After being washed with distilled water for 3 min, the sections were counter-stained with hematoxylin for 3 min, washed in distilled water for 5 min and incubated in the eosin staining solution for 3 min, dehydrated with a graded alcohol series followed by xylene, air-dried and coverslipped with neutral gum. All specimens were observed and photomicrographed under a microscope.

### Analyses of biochemical indexes in plasma

Plasma ALT, AST and TG levels were analysed using an automatic biochemical analyser (Olympus AU2700) by Nanjing General Hospital of Nanjing Military Command (Nanjing, China).

### Radioimmunoassay for plasma cortisol and TNF-α

Plasma cortisol and TNF-α concentrations were measured in duplicates using a commercially available 125I-RIA kit (Technology Research Institute of the Northern biological Inc., Beijing, China). Cross-reactivities of antibody used to any potentially competing plasma steroids of the kits were lower than 10 %. The assay was validated for use with porcine plasma. Sensitivity of the cortisol assay was 2 ng/mL, and the TNF-α kit was 6 fmol/mL respectively.

### Real-time RT-PCR for mRNA quantification

Total RNA was isolated from liver samples using TRIzol Reagent (no. 15596026, Invitrogen) according to the manufacturer’s instruction. Total RNA extracts were then treated with DNase I (D2215, Takara) to eliminate possible contamination of genomic DNA. Two micrograms of total RNA were reverse transcribed and 2 μL of diluted cDNA (1:20) were used for real-time PCR analysis. Peptidylprolyl isomerase A (PPIA) was chosen as a reference gene. All primers were synthesized by Generay Biotech and listed in Table 2. The method of 2^-ΔΔCt^ was used to analyse the real-time PCR data.

**Table 2:** Primer sequences used in real-time PCR analysis and ChIP assay

Target gene	Sequence (F: forward, R: reverse)	GenBank access	Applications
TLR2	F: GACACCGCCATCCTCATTCT	NC_010450	PCR
	R: CTTCCCGCTGCGTCTCAT		
TLR4	F: TCTACATCAAGTGCCCCTAC	NR_024169.1	PCR
	R: TAAATTCTCCCAAAACCAAC		
TNF-α	F:CCACGCTCTTCTGCCTACTGC	JF831365.1	PCR
	R:TCGGCTTTGACATTGGCTACAA		
NF-κB	F:GGGGACTACGACCTGAATGC	EU399817.1	PCR
	R:CACGGTTGTCAAAGATGGG		
IL-1α	F:TACTGACTATGGCTACCAA	NM_214029.1	PCR
	R:ATTCCAGCTGCTATTGTG		
ACC-1	F:GGCCATCAAGGACTTCAACC	NM_001114269.1	PCR
	R:ACGATGTAAGCGCCGAACTT		
FAS	F: GTCCTGCTGAAGCCTAACTC	EF589048	PCR
	R: TCCTTGGAACCGTCTGTG		
SCD-1	F: CCCAGCCGTCAAAGAGAA	NM_213781	PCR
	R: CGATGGCGTAACGAAGAAA		
PPAR-γ	F: GCCCTTCACCACTGTTGATT	NM_138711	PCR
	R: GAGTTGGAAGGCTCTTCGTG		
C/EBP-β	F: GACAAGCACAGCGACGAGTA	NM_001199889	PCR
	R: AGCTGCTCCACCTTCTTCTG		
HSL	F: ACCCTCGGCTGTCAACTTCTT	AY686758	PCR
	R: TCCTCCTTGGTGCTAATCTCGT		
ATGL	F: ACCTGTCCAACCTGCTGC	EF583921	PCR
	R: GCCTGTCTGCTCCTTTATCCA		
CPT-1α	F:ACAACGAGGTCTTCCGAT	NM_001129805.1	PCR
	R:AACGCAAAACCACCAAACCC		
ND1	F: TCCTACTGGCCGTAGCATTCCT	KF888634.1	PCR
	R: TTGAGGATGTGGCTGGTCGTAG		
ND2	F: ATCGGAGGGTGAGGAGGGCTAA	KF888634.1	PCR
	R: GTTGTGGTTGCTGAGCTGTGGA		
ND3	F: AGCACGCCTCCCATTCTCAAT	KF888634.1	PCR
	R: TGCTAGGCTTGCTGCTAGTAGG		
ND4	F: TCGCCTATTCATCAGTAAGTCA	KF888634.1	PCR
	R: GGATTATGGTTCGGCTGTGTA		
ND4L	F: GATCGCCCTTGCAGGGTTACTT	KF888634.1	PCR
	R: CTAGTGCAGCTTCGCAGGCT		
ND5	F: CGGATGAGAAGGCGTAGGAA	KF888634.1	PCR
	R: GCGGTTGTATAGGATTGCTTGT		
ND6	F: ACTGCTATGGCTACTGAGATGT	KF888634.1	PCR
	R: CTTCCTCTTCCTTCAACGCATA		
COX1	F: TGGTGCCTGAGCAGGAATAGTG	KF888634.1	PCR
	R: ATCATCGCCAAGTAGGGTTCCG		
COX2	F: GCTTCCAAGACGCCACTTCAC	KF888634.1	PCR
	R: TGGGCATCCATTGTGCTAGTGT		
COX3	F: GGCTACAGGGTTTCACGGGTTG	KF888634.1	PCR
	R: TCAGTATCAGGCTGCGGCTTCA		
ATP6	F: ACTCATTCACACCCACCACACA	KF888634.1	PCR
	R: CCTGCTGTAATGTTGGCTGTCA		
ATP8	F: TGCCACAACTAGATACATCC	KF888634.1	PCR
	R: GCTTGCTGGGTATGAGTAG		
CYTB	F: CTGAGGAGCTACGGTCATCACA	KF888634.1	PCR
	R: GCTGCGAGGGCGGTAATGAT		
PPIA	F: TCCTCCTTGGTGCTAATCTCGT	NM_214353.1	PCR
	R: TGATCTTCTTGCTGGTCTT		
ACC-1_promoter	F: ACAGCATACGGAGTTCCTGG		ChIP
	R: TGTCTTCTGCAACAATGGGA		

### Western blotting for protein quantification

Liver samples were homogenized in RIPA buffer (50 mM Tris–HCl pH 7.4, 150 mM NaCl, 1 % NP40, 0.25 % Na-deoxycholate, 1 mM PMSF, 1 mM sodium orthovanadate with Roche EDTA-free complete mini protease inhibitor cocktail, no. 11836170001). Protein concentrations were determined with a Pierce BCA Protein Assay kit (no. 23225, Thermo). Western-blot analysis for target proteins was carried out according to the protocols provided by the primary antibody suppliers. β-actin was selected as loading control. Anti-CCAAT/enhancer-binding protein-β (C/EBP-β) antibody (sc-150×, Santa Cruz, 1:200) was purchased from Santa Cruz Biotechnology; anti-SCD-1 antibody (ab39969, abcam, 1:1000) was purchased from abcam; anti-ACC-1 ntibody (BS1377, Bioworld, 1:500); anti-Peroxisome proliferator-activated receptor-γ (PPAR-γ) antibody (MB0080, Bioworld, 1:500); anti-CPT-1α antibody (BS7744, Bioworld, 1:500) and anti-β-actin antibody (AP0060, Bioworld, 1:10,000) was purchased from Bioworld. Chemiluminescent substrate (ECL) kit (34080, Pierce) was used to visualize protein bands of interest. ECL signal intensities were quantified using a VersaDoc MP 4000 system (BioRad). The content of detected proteins was presented as the fold change relative to the average content of the control group.

### Enzyme assay for mitochondrial complex IV and V activities

Liver mitochondria were isolated following previously published protocols [37] with minor modifications. Two hundred micrograms of chopped liver samples was homogenized in buffer (0.1 M Tris–MOPS, 0.1 M EGTA/Tris, and 1 M sucrose with protease inhibitor cocktail (11697498001, Roche), pH 7.4), with 12 strokes of a loose pestle in a glass Dounce homogenizer. The homogenates were centrifuged at 600 g for 10 min at 4 °C, and supernatant was collected for further centrifugation (7000 g). The pellet was then resuspended in isolation buffer and centrifuged at 7000 g for 10 min at 4 °C. The final washed mitochondrial pellet was dispersed in isolation buffer and stored at −70 °C until assayed. All the operations were carried out on ice. Mitochondrial complex IV and V activities were determined according to the instructions of commercial kits (GMS50010, GMS50083, Genmed Scientifics, Inc.).

### Chromatin immunoprecipitation (ChIP) assay

ChIP analysis was performed according to our previous publication [38] with some modifications. Briefly, 200 mg of frozen liver samples were ground in liquid nitrogen and washed with phosphate-buffered saline containing protease inhibitor cocktail (11697498001, Roche). After cross-linking in 1 % formaldehyde, the reaction was stopped with 2.5 M glycine. The pellets were washed with PBS and lysed in SDS lysis buffer containing protease inhibitors. The lysates were sonicated on ice to yield DNA fragments of 200 to 500 bp in length. After pre-clearance of the resulting chromatin with salmon sperm DNA-protein A/G agarose (50 % slurry), the immunoprecipitation was performed with 2 mg of a specific GR antibody (sc-1004×, Santa Cruz) or normal control IgG (12–370, Millipore) overnight at 4 °C. DNA fragments were then released by reverse cross-linking from the immunoprecipitated complex at 65 °C overnight. The DNA fragments were then treated with Proteinase K (Sunshine, China) at 45 °C for 1 h. Finally, the DNA was purified and resuspended in 100 μl TE buffer (10 mM Tris–HCl, 0.1 mM EDTA, pH 8.0). 2 μl of the immunoprecipitated DNA were used as a template for real-time PCR detection. ChIP results were calculated relative to the control input DNA and presented as the fold change relative to the average value of the control group. The primer is listed in Table 2. The relative GR binding was calculated using the 2^-ΔΔCt^ method.

### Statistical analysis

All statistical analyses were performed with SPSS 18.0 for Windows. All data were expressed as the mean ± SEM, and one-way ANOVA was used to assess effects. The level of significance was set at *P* < 0.05 for all analyses.

## Abbreviations

ACC-1: Acetyl-CoA carboxylase-1
ALT: Alanine aminotransferase
AST: Aspartate aminotransferase
ATGL: Adipose TG lipase
ATGL: Adipose triglyceride lipase
ATP6: ATP synthase F0 subunit 6
ATP8: ATP synthase F0 subunit 8
CEBP-β: CCAAT/enhancer-binding protein-β
ChIP: Chromatin immunoprecipitation
COX1: Cytochrome C oxidase subunit 1
COX2: Cytochrome C oxidase subunit 2
COX3: Cytochrome C oxidase subunit 3
CPT-1α: Carnitine palmitoyltransferase-1α
CYTB: Cytochrome b
ECL: Chemiluminescent substrate
FAS: Fatty acid synthase
GR: Glucocorticoid receptor
GRE: Glucocorticoid receptor element
HSL: Hormone-sensitive lipase
HSL: Hormone-sensitive lipase
IL-1α: Interleukin-1α
LPS: Lipopolysaccharide
ND1: NADH dehydrogenase subunit 1
ND2: NADH dehydrogenase subunit 2
ND3: NADH dehydrogenase subunit 3
ND4: NADH dehydrogenase subunit 4
ND4L: NADH dehydrogenase subunit 4 L
ND5: NADH dehydrogenase subunit 5
ND6: NADH dehydrogenase subunit 6
NF-κB: Nuclear factor kappa B
PPAR-γ: Peroxisome proliferator-activated receptor-γ
PPIA: Peptidylprolyl isomerase A
SCD-1: Stearoyl-CoA desaturase-1
TG: Triglyceride
TLR2: Toll-like receptor 2
TLR4: Toll-like receptor 4
TNF-α: Tumor necrosis factor-α
